# Validation of a short form of the perceptions of interparental conflict in childhood

**DOI:** 10.3389/fpsyg.2025.1518064

**Published:** 2025-08-08

**Authors:** Luciana Lassance, Alison Paradis, Natacha Godbout

**Affiliations:** ^1^Laboratory for the Study of the Well-Being of Families and Couples, Department of Psychology, Université du Québec à Montréal, Montreal, QC, Canada; ^2^Trauma and Couples Research and Intervention Unit, Department of Sexology, Université du Québec à Montréal, Montreal, QC, Canada

**Keywords:** Interparental conflict, parents, French-Canadian validation, childhood interpersonal trauma, validation

## Abstract

Exposure to interparental conflict (IC) in childhood has been documented to be an important risk factor for parents’ wellbeing and the intergenerational transmission of trauma. However, there is no French instrument available for measuring the childhood exposure to IC in parents. This study aimed to assess the psychometric properties of a short form of the Perceptions of Interparental Conflict questionnaire (PIC-SF), which assesses memories of interparental conflict that occurred in the parents’ own childhood. Data were collected between August 2021 and February 2023, and participants had a mean age of 39.2 years old (*SD* = 5.4). Exploratory and Confirmatory factor analyses were conducted among a representative sample of 610 parents of a toddler in the province of Québec (Canada), which was split into two subsamples (*n* = 305). Results indicate that the PIC-SF demonstrates strong internal consistency supported by Cronbach’s alpha (*α* = 0.95) and McDonald’s omega (*ω* = 0.95). Additionally, a unifactorial structure was supported, accounting for 74% of the variance. Correlation analyses indicated that memories of exposure to IC in childhood were related to childhood interpersonal trauma, increased psychological distress, self-capacity alterations, and destructive conflict management strategies. Researchers and practitioners have access to a promising measure of memories of exposure to IC in childhood that could be used, free of fees, to document past family experiences and inform well-tailored services.

## 1 Introduction

Exposure to interparental conflict (IC) in childhood refers to witnessing dysfunctional behaviors during parental conflict (e.g., yelling, complaining, or being mean to each other) in a typical year before the age of 18. Such exposure often reflects a broader pattern of dysfunctional family dynamics, where conflict behaviors indicate deeper, ongoing relational difficulties within the family system (van Eldik et al., 2020). Although the prevalence and degree of adults exposed to IC in childhood are hard to specify, 36 to 40% of the general population report having been exposed to psychological violence in childhood (Dugal et al., 2019). Considering that IC does not always imply violence, these rates highlight the extent of the phenomenon in the population.

Parenting a young child is associated with considerable stress in the lives of individuals and couples, marked by significant structural changes and increased demands on the physical, psychological, and material resources of parents (Ensink et al., 2017). Parents must adapt to new realities and constantly meet the needs of their children, while giving less time, energy or importance to their couple relationship (Ensink et al., 2017). Moreover exposure to IC in childhood is a factor that may increase vulnerability to stress, as it may alter how the individual reacts to it, eventually leading to an increased sensitivity to stress (Biaggi and Pariante, 2015). As a result, parents may experience more distress, have more conflicts with their partners, and behave more negatively toward their children.

Studies have indicated a small to moderate correlation between exposure to IC in childhood and poorer interpersonal functioning throughout the lifespan (Kumar and Mattanah, 2018), depression and anxiety symptoms, difficulties in regulating negative emotions (e.g., anger, sadness), maladaptive coping strategies (Siffert and Schwarz, 2011), and intergenerational transmission of trauma (Kopystynska et al., 2022). Empirical studies suggest that exposure to IC in childhood is specifically associated with a lower ability to manage disagreements with the romantic partner once in adulthood (Cui et al., 2008). Adults who have been exposed to destructive conflict management strategies between their parents report a higher frequency of arguments and more hostile behaviors during conflicts with their romantic partners compared to those who have not been exposed (Cui et al., 2008). Chiesa et al.’s (2018) review of the literature suggests a potential past interparental conflict spillover on parenting, with higher levels of exposure to IC in childhood being associated with parental physical aggression and neglect.

Considering the negative impact exposure to IC in childhood may have on parents’ wellbeing and on the intergenerational transmission of trauma, it is justified to validate an instrument that may help diagnose this problem in the parents of the current generation. Most instruments measuring exposure to IC in childhood were validated with children and adolescents and adapted to be used retrospectively in adults. Moreover, it is important to validate a single instrument that is effective for both mothers and fathers, as it has been demonstrated that girls and boys may have experienced exposure to IC differently (Kret and De Gelder, 2012). Thus, a validated measure adapted to adults is needed if only for preventative purposes.

Well-validated scales were developed to measure exposure to IC in children, including the Children’s Perceptions of Interparental Conflict Scale (CPIC; Grych et al., 1992); a 49-item self-reported questionnaire measuring the frequency and intensity of children’s exposure to interparental conflict, as well as their distress levels and coping strategies related to it. Kline and colleagues adapted this measure into the Perceptions of Interparental Conflict (PIC; Kline et al., 2003) to be used in a retrospective way by young adults. The PIC is a 13-item self-reported questionnaire assessing the frequency and intensity dimensions of the original version, as studies reported them as being the most related to child adjustment and presenting the highest internal consistency. Validated in a sample of young adults (*M_age_* = 20 years), the PIC includes items assessing memories of dysfunctional behavior between parents during conflicts (e.g., yelling, complaining, being mean to each other). Factor analysis revealed a single factor structure, although factor loading values have not been reported. The instrument showed strong internal consistency (*α* = 0.95) in adult samples (e.g., Dennison et al., 2014), supporting the unidimensional structure suggested by factor analysis and high inter-item correlations. However, this instrument has not yet been validated in French nor in parents, preventing from studying potential intergenerational transmission of conflicts between parents.

The purpose of this study was to assess the psychometric properties of a short form of the PIC (PIC-SF) by examining the factor structure and psychometric properties in one subsample and confirming the structure in a second one. It was expected that the factor structure of the PIC-SF would be unidimensional, present satisfactory internal consistency, and that higher scores on the PIC-SF would be correlated with higher psychological distress, childhood interpersonal trauma, self-capacities impairment, as well as more destructive conflict management strategies.

## 2 Method

### 2.1 Development of a short form of the perceptions of interparental conflict scale with adults

To develop a short form of the PIC, a team of family violence experts reviewed the construct content of the original items to evaluate the feasibility of creating a more concise version. The goal was to make the instrument easier to integrate in large survey and reduce the time burden for parents. First, the team decided to exclude the four reverse-scored items (i.e., “I never saw my parents arguing or disagreeing,” “My parents hardly ever argue”), as they were conceptually redundant with more directly worded items (i.e., “I often saw my parents arguing “, “My parents argued or disagreed a lot”). Reverse-scored items can introduce interpretive issues and may negatively impact the scale’s psychometric properties (Tsang et al., 2017). Second, two questions measuring more extreme levels of conflict (i.e., “my parents have broken or thrown things during arguments”; “my parents have pushed or shoved each other during arguments”) were combined into one simpler question that measures the presence of minor physical violence in a conflict between parents (i.e., “My parents have pushed/shoved each other or broken/thrown things during an argument”; Straus and Douglas, 2004). Moreover, in the original article, these items showed the lowest item-to-total correlations (0.46 and 0.42, respectively) and also loaded onto a different factor. To reduce redundancy and enhance the psychometric coherence of the scale, we combined them into a single item reflecting overt physical conflict. Lastly, items were translated from English into French using the back-translation procedure (Vallerand, 1989) with a committee of bilingual researchers ascertaining correspondence and adaptation. The original English version was first translated into French and reviewed by a committee of bilingual researchers. A bilingual research assistant, who had not seen the original instrument, back-translated the items into English. The original and back-translated English versions were then compared to assess accuracy and cultural adaptation. The committee of bilingual researchers deemed the final French translation satisfactory. Thus, the questionnaire was reduced from 13 items to 8 items in the final version, resulting in a shorter, more concise version suitable for use in research and practice.

### 2.2 Participants and procedure

This study is part of wave 4 from a larger project including several measurement waves, which investigates the mental and relational health of parents of an infant in Québec. Parents were randomly selected from the birth records of the Québec Parental Insurance Plan list (QPIP) and selected according to the following inclusion criteria: (1) being parent of an infant under six-months-old, (2) being 18 years of age or older, and (3) reading and understanding French fluently. Parents’ contact information was provided by the QPIP. Participants were reached from August 2021 to February 2023 by e-mail with a telephone follow-up and invited to individually complete a questionnaire on Qualtrics Internet platform. Participation was voluntary and parents were provided with information regarding the study’s purpose, the measures taken to ensure confidentiality and security of their data. Each participant was offered a $20 gift card for his or her participation. Both parents were invited to participate in the study even if their partner declined the invitation. For these parents whose partners also participated, only one member of each couple was randomly selected to respect data independence (Kenny and Judd, 1986). In the present study, children are aged from 28 to 34 months. The project was approved by the institution’s human research ethics committee at University of Quebec in Montreal. A sample of 610 Québec French-Canadian participants was initially screened for insufficient effort responding using intra-individual response variability (IRV) calculated on the PIC-SF. The mean IRV was 0.82 (*SD* = 0.55), and participants with IRV scores exceeding 1.92 (i.e., 2 *SD* above the mean) were flagged for further review, as such scores may indicate highly inconsistent responding. Four participants (0.7%) met this criterion. To assess the validity of their responses, their patterns on other questionnaires included in the survey were examined. These participants exhibited adequate variability across measures, suggesting that the high IRV on the PIC-SF reflected a genuine response style. As a result, no cases were excluded. The full sample was then randomly divided into two equal subsamples. Sociodemographic information for each subsample is presented in Table 1.

**Table 1:** Sociodemographic characteristics of participants.

Characteristics	Subsample 1		Subsample 2	
	*n*	%	*n*	%
Gender				
Self-identified as mothers	170	55.7	179	58.7
Self-identified as fathers	135	44.3	126	41.3
Sexual orientation				
Heterosexual	303	99.3	298	97.7
Homosexual or questionning	2	0.6	7	2.3
Marital status				
Common-law relationship	212	73.8	200	69
Married	75	24.6	90	31
Occupation				
Work full-time	221	72.5	222	72.8
Work part-time	24	7.9	29	9.5
Studying	7	2.3	13	4.3
Parental leave/ stay-at-home parents	50	16.4	35	11.5
Unemployed/medical leave	3	0.9	6	1.9
Place of birth				
Canada	264	86.6	252	82.6
Western Europe	13	4.3	16	5.2
Eastern Europe	4	1.3	6	2
Africa	9	3	19	6.2
Asia	3	1	1	0.3
Middle East	3	1	2	0.7
Latin / South America	7	2.3	8	2.6

	*M*	*SD*	*M*	*SD*
Age				
Mothers (years)	37.9	4.8	38.1	4.6
Fathers (years)	40.9	5.6	40.8	6.0
Number of children	2.1		2.1	
Relationship duration (years)	9.3	4.2	9.6	4.1
Family median income ($ CAN)	139,999		139,999	

### 2.3 Measures

#### 2.3.1 Perception of interparental conflict in childhood

Exposure to IC in childhood was measured using the 8-item PIC-SF. Items are answered on a 6-point Likert scale ranging from 1 = strongly disagree to 6 = strongly agree. A global score was computed by averaging responses across items.

#### 2.3.2 Childhood interpersonal trauma

Six types of Childhood interpersonal trauma (i.e., exposure to physical and psychological interparental violence, and psychological and physical neglect and abuse) were measured using the French version of the Childhood Cumulative Trauma Questionnaire (Godbout et al., 2017). Participants were asked to answer on a 6-point Likert scale (ranging from 0 = never to 5 = every day or almost) how often they had experienced these types of interpersonal trauma in a typical year before age 18. Each type of interpersonal trauma is measured by averaging the items of its respective subscale. Physical violence scale is composed of five items (*α* = 0.80) and Psychological violence scale is composed of three (*α* = 0.60). Physical neglect scale is composed of two items (for which Cronbach alpha coefficients could not be computed; Eisinga et al., 2013). Psychological neglect scale is composed of three items (*α* = 0.80) while Exposure to physical and psychological interparental violence are measured by one question each one. This questionnaire also allows us to compute a cumulative score of childhood interpersonal trauma by dichotomizing each form of Childhood interpersonal trauma as experienced (1) or not (0), and a sum is used to obtain a continuous score of cumulative childhood interpersonal trauma, ranging from 0 = no childhood trauma to 6 = six childhood traumas. These types of trauma were included in the cumulative index score only if they had occurred at least once in a typical year before the age of 18. Past findings indicated that the cumulative score is the best predictor of outcomes (Finkelhor et al., 2007). This score was also used in this study.

#### 2.3.3 Psychological distress

Psychological distress was measured using the French version of the 6-item Kessler Psychological Distress Scale (Kessler et al., 2003; Sampasa-Kanyinga et al., 2018) on a 5-point Likert scale ranging from 0 = never to 4 = always (*α* = 0.85). Total score was calculated by averaging item scores.

#### 2.3.4 Altered self-capacities

Interpersonal conflict (IC), Identity impairment (II) and affect dysregulation (AD) were measured using the French version of the Inventory of Altered Self-Capacities (Bigras and Godbout, 2020; Briere and Runtz, 2002). Each scale is composed of nine items rated on a 5-point Likert scale ranging from 1 = never to 5 = very often (*α*_IC_ = 0.84; *α*_II_ = 0.88; *α*_AD_ = 0.90). Total scores were obtained by averaging item scores.

#### 2.3.5 Destructive conflict management strategies

Destructive management strategies adopted by partners during a conflict were measured using 12 items of the French version of the Conflict Resolution Styles Inventory (Fortin et al., 2020; Kurdek, 1994). Parents were asked to answer how often they use different behaviors during conflicts with their partners on a 5-point Likert scale from 1 = never to 5 = always (*α* = 0.83). Total scores were obtained by averaging item scores.

## 3 Results

This study was conducted in accordance with the Standards for Educational and Psychological Testing (AERA, APA and NCME, 2014) with attention to internal structure, reliability, score comparability between mothers and fathers as well as testing invariance of the structure.

### 3.1 Descriptive analyses

Item means, standard deviations, skewness and kurtosis are presented in Table 2. In both subsamples, the response trend was concentrated in the options “Strongly Disagree” and “Disagree,” which is congruent with the positive skewness distribution observed in all items (for more details, see Supplementary Table S1). This was expected, since the PIC-SF measures extreme behaviors in a non-clinic population. Standard deviations across items are very similar in both subsamples, indicating consistent variability across items (SS1: SD_max_/SD_min_ = 1.3; SS2: SD_max_/SD_min_ = 1.4). In Subsample 1, the mean score of the PIC-SF was 2.6 (*SD* = 1.3). Mothers reported significantly higher levels on PIC-SF scores (*t*_(303)_ = 2.29, *p* = 0.023; *M* = 2.7, *SD* = 1.4) than fathers (*M* = 2.4, *SD* = 1.2), with a small effect size (*r* = 0.13). In Subsample 2, the mean score of the PIC-SF was 2.5 (*SD* = 1.4), with no significant difference (*t*_(303)_ = 1.37, *p* = 0.173) between mothers (*M* = 2.6, *SD* = 1.4) and fathers (*M* = 2.4, *SD* = 1.2) and a small effect size (*r* = 0.08).

**Table 2:** Means, standard deviations, and factor loadings from exploratory and confirmatory factor analysis.

Items	Subsample 1 (EFA)					Subsample 2 (CFA)				
	*M*	*SD*	*S*	*K*	*ß*	*M*	*SD*	*S*	*K*	*ß*
My parents would get really mad when they argued [*Mes parents devenaient vraiment fâchés quand ils se disputaient*]	3.1	1.6	0.20	−1.19	0.85	3.2	1.7	0.14	−1.30	0.83
My parents argued or disagreed a lot [*Mes parents se disputaient ou étaient souvent en désaccord*]	2.9	1.6	0.40	−1.05	0.87	2.9	1.6	0.37	−1.14	0.88
My parents were often mean to each other even when I was around [*Mes parents étaient souvent méchants l’un envers l’autre, même quand j’étais là*]	2.2	1.5	1.09	0.07	0.91	2.2	1.5	1.09	0.04	0.86
I often saw my parents arguing [*J’ai souvent vu mes parents se disputer*].	2.9	1.7	0.45	−1.09	0.91	2.8	1.7	0.55	−0.97	0.91
When my parents argued, they would say mean things to each other [*Quand mes parents se disputaient, ils se disaient des choses méchantes*].	2.4	1.6	0.98	−0.24	0.91	2.3	1.6	0.96	−0.39	0.89
When my parents argued, they would yell a lot [*Quand mes parents se disputaient, ils criaient beaucoup*].	2.7	1.7	0.63	−0.86	0.91	2.6	1.7	0.68	−0.82	0.91
My parents often nagged and complained about each other [*Mes parents chialaient et se plaignaient souvent l’un de l’autre*].	2.6	1.6	0.71	−0.78	0.82	2.4	1.6	0.81	−0.68	0.82
My parents have pushed/shoved each other or have broken/thrown things during arguments [*Mes parents se sont déjà poussés/ bousculés ou ont cassé/lancé des objets au cours d’une dispute*].	1.7	1.3	2.14	3.50	0.69	1.6	1.2	2.43	4.85	0.59
Cronbach’s alpha (*α*)		0.95 (95% CI [0.94, 0.96])				0.95 (95% CI [0.94, 0.96])				
McDonald omega (*ω*)		0.95 (95% CI [0.94, 0.96])				0.95 (95% CI [0.94, 0.96])				

*S, skewness; K, kurtosis.

### 3.2 Exploratory factor analysis (EFA)

EFA was conducted on the 8 items of the PIC-SF in Subsample 1. Results revealed a unidimensional model explaining 74% of the variance (see Table 2 for details). Parallel analysis and a minimum average partial test were conducted to determine the number of factors to retain (O’Connor, 2000), which confirmed the unifactorial structure (see Supplementary Table S2). The tight interitem correlations (see Table 3), alongside the identical values for Cronbach’s alpha [*α* = 0.95, 95% CI (0.94, 0.96)] and McDonald (*ω* = 0.95, 95% CI [0.94, 0.96]) also guarantee the unidimensionality of the questionnaire (*α* ≥ 0.70; Nunnally, 1978). The PIC-SF also showed a low standard error of measurement (SEM = 0.29) suggesting good accuracy.

**Table 3:** Correlation matrix for Subsample 1 and Subsample 2.

Items	1	2	3	4	5	6	7	8
1. My parents would get really mad when they argued	-	0.78	0.68	0.74	0.71	0.78	0.64	0.49
2. My parents argued or disagreed a lot	0.75	-	0.75	0.84	0.75	0.80	0.69	0.51
3. My parents were often mean to each other even when I was around	0.70	0.75	-	0.76	0.86	0.73	0.77	0.57
4. I often saw my parents arguing	0.76	0.83	0.77	-	0.78	0.87	0.72	0.51
5. When my parents argued, they would say mean things to each other	0.75	0.73	0.86	0.78	-	0.79	0.79	0.54
6. When my parents argued, they would yell a lot	0.80	0.75	0.77	0.82	0.83	-	0.71	0.57
7. My parents often nagged and complained about each other	0.56	0.71	0.71	0.72	0.69	0.66	-	0.41
8. My parents have pushed/shoved each other or have broken/thrown things during arguments	0.49	0.43	0.62	0.51	0.60	0.59	0.53	-

Correlation coefficients for Subsample 1 are presented below the diagonal, and those for Subsample 2 are presented above the diagonal.

### 3.3 Confirmatory factorial analysis (CFA)

CFA was conducted on Mplus v8.5 (Muthén and Muthén, 2017) on the 8 items of the PIC-SF in Subsample 2. Items were treated as continuous and the robust maximum likelihood estimator was used, as it considers the non-normality of the data distribution. Results revealed satisfactory model fit for a single factor structure, χ^2^(20) = 131.91; *p* < 0.001; CFI = 0.93; TLI = 0.90; SRMR = 0.03, but a RMSEA slightly above optimal cutoff; RMSEA = 0.14, 90% CI [0.11; 0.16]. This was to be expected since, especially in simple CFAs (i.e., single-factor structure); this index penalizes models with few degrees of freedom even when the model provides a good representation of the data (Kenny et al., 2015). Similarly, the χ^2^ is expected to be significant in large samples (Caron, 2018). For this reason, decision on the acceptance of the model was based on the other fit indices (i.e., CFI, TLI and SRMR) which supported good model fit. Factor loading coefficients ranged from 0.59 to 0.91 (see Table 2) and deemed satisfactory. Both Cronbach’s alpha [*α* = 0.95; 95% CI (0.94, 0.96)] and McDonald’s omega [*ω* = 0.95; 95% CI (0.94, 0.96)] reached the same high value, further confirming the instrument’s strong internal consistency and supporting the unidimensional structure of the PIC-SF. The accuracy of the instrument was also supported in this sample (SEM = 0.31). Then, invariance analysis was conducted in order to test the factorial structure of the instrument according to gender, following Byrne’s and Chen’s recommendations (Chen, 2007; Byrne, 2012). Results confirm a total gender invariance at the configural, metric and structural levels (see Supplementary Table S3).

### 3.4 Convergent and divergent validity

Convergent and divergent validity were measured using Pearson correlation with confidence intervals in both subsamples. Effect sizes were interpreted using two sets of empirically derived benchmarks: Gignac and Szodorai (2016) defined correlations of *r* = 0.10 as relatively small, 0.20 as typical, and 0.30 as relatively large; Lovakov and Agadullina (2021) proposed slightly higher thresholds, with *r* = 0.12 considered small, 0.24 medium, and 0.41 large. Correlations presented small to large effects, indicating distinct concepts related in the expected ways in both subsamples (i.e., *r_ss1_* = 0.19 to 0.62, *r_ss2_* = 0.13 to 0.66; see details in Table 4). Confidence intervals provided additional evidence for the strength and precision of these associations. The PIC-SF score was associated with higher psychological distress, self-capacities disturbances (affect dysregulation, identity impairment, and interpersonal conflicts), childhood interpersonal trauma (psychological and physical neglect and abuse, exposure to interparental violence, and cumulative trauma), and a greater use of destructive conflict management strategies. Fisher’s *Z*-transformation test showed that correlations were not significantly different between the two subsamples (see details in Table 4).

**Table 4:** Convergent and divergent validity of the PIC-SF.

Measures	Range	*M (SD)*		Pearson coefficient [95%CI]		Fisher’s *Z*-test	
		SS1	SS2	SS1	SS2	z	*p*
PIC-SF	1–8	2.6 (1.3)	2.5 (1.4)	-	-		
Childhood interpersonal trauma							
Psychological neglect	0–5	0.9 (1.1)	0.9 (1.1)	0.40^**^[0.30, 0.49]	0.42^**^[0.32, 0.51]	−0.29	0.385
Physical neglect	0–5	0.2 (0.7)	0.1 (0.5)	0.32^**^[0.21, 0.42]	0.34^**^[0.24, 0.44]	−0.27	0.392
Psychological abuse	0–5	0.4 (0.9)	0.4 (0.8)	0.36^**^[0.26, 0.45]	0.41^**^[0.32, 0.51]	−0.86	0.194
Physical abuse	0–5	0.2 (0.5)	0.2 (0.5)	0.27^**^[0.16, 0.37]	0.33^**^[0.23, 0.43]	−0.81	0.210
Exposure to psychological interparental violence	0–5	0.9 (1.4)	1.0 (1.5)	0.62^**^[0.55, 0.69]	0.66^**^[0.59, 0.72]	−0.83	0.204
Exposure to physical interparental violence	0–5	0.2 (0.8)	0.1 (0.6)	0.34^**^[0.23, 0.43]	0.31^**^[0.20, 0.41]	0.41	0.341
Cumulative childhood interpersonal trauma	0–6	2.0 (1.6)	2.0 (1.6)	0.49^**^[0.39, 0.57]	0.57^**^[0.49, 0.64]	−1.36	0.087
Psychological distress	0–4	0.9 (0.7)	0.9 (0.7)	0.27^**^[0.16, 0.37]	0.18^**^[0.07, 0.29]	1.17	0.122
Altered self-capacities							
Interpersonal conflict	1–5	1.7 (0.6)	1.7 (0.5)	0.19^**^[0.08, 0.29]	0.26^**^[0.15, 0.36]	−0.90	0.183
Identity impairment	1–5	1.5 (0.6)	1.6 (0.6)	0.25^**^[0.14, 0.35]	0.13^**^[0.02, 0.24]	1.53	0.063
Affect dysregulation	1–5	1.6 (0.7)	1.5 (0.6)	0.30^**^[0.20, 0.40]	0.19^**^[0.08, 0.30]	1.44	0.075
Destructive conflict management strategies	1–5	1.7 (0.5)	1.7 (0.5)	0.24^**^[0.12, 0.35]	0.18^**^[0.06, 0.29]	0.71	0.240

SS1, Subsample 1; SS2, Subsample 2.

*p < 0.05; **p < 0.01.

## 4 Discussion

This study aimed to validate the PIC-SF within a French-Canadian sample of parents. Results confirmed a unidimensional structure and demonstrated strong internal consistency, supported by Cronbach’s alpha, McDonald’s omega and high inter-item correlations. These results ensure that the questionnaire provides a solid and unambiguous retrospective assessment of adults’ memories of exposure to IC in childhood. The combination of high internal consistency and low standard measurement error suggests that the PIC-SF yields scores with good precision and reduced measurement error. These characteristics support the instrument’s accuracy in assessing perceived IC. With low standard error of measurement, the PIC-SF ensures stable and consistent measurements, which enhances its ability to provide precise and accurate data for both group-or individual-level assessments.

Invariance analysis results support full measurement invariance across gender, indicating that the PIC-SF assesses exposure to IC in childhood similarly for mothers and fathers, allowing score comparison between them. Mothers’ recollections of IC in childhood were significantly higher than fathers’ levels in Subsample 1. This could reflect general gender differences in expression and interpretation of emotions, as studies revealed that girls are more skilled than boys at recognizing emotions, especially expressions of fear and sadness (Saylik et al., 2018; Kapitanović et al., 2023), as a consequence of gender-based socialization. However, this difference between mothers and fathers did not replicate in Subsample 2 and should be interpreted with caution. This could reflect the natural variability of the data, which can lead to small differences in averages between subsamples (Maxwell et al., 2015).

This study showed links between parents’ recollections of IC in their childhood and childhood interpersonal trauma (e.g., psychological and physical exposure to interparental violence, psychological and physical neglect and abuse, and cumulative trauma). These associations suggest that exposure to IC in childhood may be a sign that parents’ family-of-origin dynamic was dysfunctional, concurring with family systems theory that posits that the interparental relationship can affect the course of parent–child relationships (Cowan and Cowan, 2015). Parents who reported being exposed to IC in childhood may have undergone negative parenting, as previous studies showed that witnessing higher conflicts among their parents is related to history of parents who were less psychologically and physically available to their needs (Kopystynska et al., 2022). Associations between parents’ recollections of IC in their childhood and interpersonal conflicts suggest that experiencing interparental conflicts in childhood may affect one’s capacity to regulate its own emotions and to connect with others. Associations between parents’ recollection of IC in their childhood and altered self-capacities suggest that experiencing interparental conflicts in childhood may affect one’s view of self as well as his capacity to regulate its own emotions and to connect with others. The lack of a secure environment may sensitize children to interpersonal stress, hindering the internalization of a positive image of self and others as well as the learning of social and emotional regulation skills (Ford and Courtois, 2020). Consequently, these individuals are more likely to experience psychological distress when facing stressful life periods (Ford and Courtois, 2020). Higher levels of exposure to IC in childhood are significantly associated with destructive conflict management strategies in adulthood. As postulated by social learning theory (Bandura, 1977), conflict management strategies are learned in childhood through the witnessing of parents during an argument. By being exposed to destructive conflict strategies (e.g., saying hurtful words to partner), children may learn that their use is appropriate and acceptable and these learning may shape individuals’ behaviors in their own relationships as adults. The small and medium sized effects found for these associations show, however, that these variables share a small percentage of their variance. This was expected, as these variables may also be related with other factors, such as depression, past traumatic events, or marital problems (Li and Johnson, 2018; Bonache et al., 2019). These correlations did not differ significantly between the two subsamples, suggesting that the pattern of associations is stable and generalizable.

The limitations of this study need to be considered. First, PIC-SF is a self-reported and retrospective questionnaire that undoubtedly reflects memory or social desirability bias. The use of prospective longitudinal data could provide complementary information on its convergent validity. It is advisable to combine the PIC-SF with qualitative data (e.g., interviews) in order to obtain more precise information about interparental conflicts they witnessed in childhood. Second, the questionnaire was validated in a normative population of parents who reported low scores of exposure to IC in childhood. It is then important to validate it in populations diagnosed with mental health problems and experiencing relationship issues (e.g., marital distress, intimate violence), for whom exposure to IC is more frequent. This implies that correlations between PIC-SF and the other instruments are based on a small proportion of the sample and generalization to clinical populations should be made with caution. Moreover, test–retest study protocols should be conducted to establish the time reliability of the instrument.

In conclusion, the PIC-SF presents satisfactory psychometric qualities in a population of parents. It provides researchers with a short, validated, and reliable questionnaire that allows the study of the influence of family of origin on a range of psychological and relational variables in adulthood. Being shorter than previous questionnaires used to access adults’ memories of exposure to IC in childhood, this instrument can be integrated into longer surveys focused on psychological and relational health. Eventually, this instrument could supply clinicians with information on the parent’s past family problems, allowing intervention focusing on the development of coping and problem resolution skills, which could prevent the intergenerational transmission of interparental conflict.

## Data Availability

The original contributions presented in the study are included in the article/Supplementary material, further inquiries can be directed to the corresponding author/s.
