# Supplementary material for: Validation of a short form of the perceptions of interparental conflict in childhood
Source: Front Psychol. 2025 Aug 8;16:1518064. doi: 10.3389/fpsyg.2025.1518064 (PMC12372336; doi:10.3389/fpsyg.2025.1518064)
Supplement: Supplementary file 1 [file Table_1.docx]

Supplementary Material

# Supplementary Tables

| Table S1  Items Descriptive Statistics and response distribution | | | | | | | | | | | | | | | | | |
| --- | --- | --- | --- | --- | --- | --- | --- | --- | --- | --- | --- | --- | --- | --- | --- | --- | --- |
|  | Subsample 1 | | | | | | | |  | Subsample 2 | | | | | | | |
|  |  |  | Responses frequency (%) | | | | | |  | Responses frequency (%) | | | | | | | |
| Item | Mode | Range | 1 | 2 | 3 | 4 | 5 | 6 |  | Mode | Range | 1 | 2 | 3 | 4 | 5 | 6 |
|  | 4 | 5 | 21.6 | 22 | 10.5 | 23.6 | 13.1 | 9.2 |  | 1 | 5 | 23 | 19.7 | 10.8 | 20 | 16.7 | 9.8 |
|  | 1 | 5 | 24.3 | 23.6 | 15.1 | 16.1 | 12.8 | 8.2 |  | 1 | 5 | 26.2 | 23.9 | 10.8 | 18.7 | 13.8 | 6.6 |
|  | 1 | 5 | 45.7 | 24 | 9.2 | 10.2 | 7.2 | 3.6 |  | 1 | 5 | 50.2 | 20 | 6.2 | 13.4 | 5.9 | 4.3 |
|  | 2 | 5 | 23.6 | 27.5 | 12.8 | 12.8 | 14.8 | 8.5 |  | 1 | 5 | 30.2 | 22.6 | 13.8 | 13.4 | 10.8 | 9.2 |
|  | 1 | 5 | 40.7 | 25.9 | 9.5 | 10.2 | 8.2 | 5.6 |  | 1 | 5 | 48.4 | 16.4 | 10.5 | 10.5 | 8.2 | 5.9 |
|  | 1 | 5 | 35.4 | 20.3 | 12.1 | 15.4 | 8.5 | 8.2 |  | 1 | 5 | 38.3 | 19.1 | 10.2 | 16.8 | 6.9 | 8.6 |
|  | 1 | 5 | 37.8 | 20.7 | 13.2 | 10.9 | 11.5 | 5.9 |  | 1 | 5 | 43.2 | 19.5 | 9.6 | 12.5 | 10.2 | 5 |
|  | 1 | 5 | 72.9 | 12.2 | 2.6 | 5 | 3.3 | 4 |  | 1 | 5 | 77.3 | 10.7 | 1.3 | 4.3 | 3.3 | 3 |

*Note*. 1= Strongly Disagree; 2= Disagree; 3= Somewhat disagree; 4= Somewhat agree; 5= Agree; 6= Strongly Agree

| Table S2 | | | | | |
| --- | --- | --- | --- | --- | --- |
| *MAP Test and Parallel Analysis Results for the Number of Factors (Subsample 1)* | | | | | |
| N^o^ | Eigenvalues | Avarage partial correlation | | Random Data Eigenvalues | |
|  |  | Squared | Power 4 | Means | %95 |
| 0 | 5.95 | 0.50 | 0.27 | 1.25 | 1.32 |
| 1 | 0.65 | 0.05* | 0.01** | 1.15 | 1.21 |
| 2 | 0.45 | 0.09 | 0.02 | 1.09 | 1.13 |
| 3 | 0.29 | 0.13 | 0.04 | 1.02 | 1.06 |
| 4 | 0.22 | 0.19 | 0.09 | 0.97 | 1.01 |
| 5 | 0.21 | 0.27 | 0.17 | 0.91 | 0.95 |
| 6 | 0.13 | 0.44 | 0.35 | 0.85 | 0.89 |
| 7 | 0.11 | 1.00 | 1.00 | 0.77 | 0.83 |
| * *The smallest average squared partial correlation ** The smallest 4 power partial correlation* | | | | | |

| Table S3 | | | | | | | | | |  |
| --- | --- | --- | --- | --- | --- | --- | --- | --- | --- | --- |
| *Comparison fit indices between gender invariance models (Subsample 2)* | | | | | | | | | |  |
| Models | χ^2^ | *df* | CFI | RMSEA | SRMR | Δ χ^2^ | Δ*df* | ΔCFI | ΔRMSEA | ΔSRMR |
| Configural | 212.426 | 40 | .929 | .168 | .034 | - | - | - | - | - |
| Metrical | 214.884 | 47 | .931 | .153 | .037 | 2.458 | 7 | .002 | .015 | .003 |
| Scalar | 222.708 | 54 | .931 | .143 | .037 | 7.824 | 7 | .000 | .010 | .000 |
| Δ Thresholds (Chen, 2007) : Metrical invariance: ΔCFI ≤ .01, ΔRMSEA ≤ .015, ΔSRMR ≤ .03; Scalar invariance: ΔCFI ≤ .01, ΔRMSEA ≤ .015, ΔSRMR ≤ .01 | | | | | | | | | | |
